# Circadian miR-449c-5p regulates uterine Ca^2+^ transport during eggshell calcification in chickens

**DOI:** 10.1186/s12864-021-08074-3

**Published:** 2021-10-26

**Authors:** Zhifu Cui, Zhichao Zhang, Felix Kwame Amevor, Xiaxia Du, Liang Li, Yaofu Tian, Xincheng Kang, Gang Shu, Qing Zhu, Yan Wang, Diyan Li, Yao Zhang, Xiaoling Zhao

**Affiliations:** 1 Department of Animal Science, Farm Animal Genetic Resources Exploration and Innovation Key Laboratory of Sichuan Province, Sichuan Agricultural University, Apt 211, Huimin Road, Wenjiang District, Chengdu, 611130 Sichuan Province People’s Republic of China; 2 Department of Pharmacy, College of Veterinary Medicine, Sichuan Agricultural University, Chengdu, Sichuan Province People’s Republic of China

**Keywords:** Chicken uterine, Circadian miRNAs, Tubular gland cells, Ca^2+^ transport

## Abstract

**Background:**

miRNAs regulate circadian patterns by modulating the biological clocks of animals. In our previous study, we found that the clock gene exhibited a cosine expression pattern in the fallopian tube of chicken uterus. Clock-controlled miRNAs are present in mammals and *Drosophila*; however, whether there are clock-controlled miRNAs in the chicken uterus and, if so, how they regulate egg-laying rhythms is unclear. In this study, we selected 18 layer hens with similar ovipositional rhythmicity (each of three birds were sacrificed for study per 4 h throughout 24 h); their transcriptomes were scanned to identify the circadian miRNAs and to explore regulatory mechanisms within the uterus of chickens.

**Results:**

We identified six circadian miRNAs that are mainly associated with several biological processes including ion trans-membrane transportation, response to calcium ion, and enrichment of calcium signaling pathways. Verification of the experimental results revealed that miR-449c-5p exhibited a cosine expression pattern in the chicken uterus. Ca^2+^-transporting ATPase 4 (*ATP2B4*) in the plasma membrane is the predicted target gene of circadian miR-449c-5p and is highly enriched in the calcium signaling pathway. We speculated that clock-controlled miR-449c-5p regulated Ca^2+^ transportation during eggshell calcification in the chicken uterus by targeting *ATP2B4*. ATP2B4 mRNA and protein were rhythmically expressed in the chicken uterus, and dual-luciferase reporter gene assays confirmed that ATP2B4 was directly targeted by miR-449c-5p. The expression of miR-449c-5p showed an opposite trend to that of ATP2B4 within a 24 h cycle in the chicken uterus; it inhibited mRNA and protein expression of ATP2B4 in the uterine tubular gland cells. In addition, overexpression of *ATP2B4* significantly decreased intracellular Ca^2+^ concentration (*P* < 0.05), while knockdown of *ATP2B4* accelerated intracellular Ca^2+^ concentrations. We found similar results after *ATP2B4* knockdown by miR-449c-5p. Taken together, these results indicate that *ATP2B4* promotes uterine Ca^2+^ trans-epithelial transport.

**Conclusions:**

Clock-controlled miR-449c-5p regulates Ca^2+^ transport in the chicken uterus by targeting *ATP2B4* during eggshell calcification.

**Supplementary Information:**

The online version contains supplementary material available at 10.1186/s12864-021-08074-3.

## Background

Animal physiology is dependent on circadian clocks [1–3] located in peripheral tissues for the maintenance of temporal order [4–7]. The master clock is located in the suprachiasmatic nucleus (SCN) and many peripheral tissues involved in these clock cycles are known as oscillators [4–7]. Specific oscillators associated with circadian clocks are categorized as circadian oscillators [8]. Expression of these circadian oscillators take place within an approximately 24 h period, which ultimately forms the circadian biological clock [8]. Molecular clockworks modulate circadian rhythms in every cell that is controlled by circadian genes and proteins via transcriptional-translational feedback loop circulation [9, 10]. Previous studies identified CLOCK and BMAL1 as basic helix-loop-helix (bHLH)-containing transcription factors and they play important roles in the oscillator loops [11–14]. For instance, the CLOCK-BMAL1 complex is found in the mammalian circadian clock, where it binds to the CACGTG E-box or its allied E-box-like sequence to promote rhythmic genes, regulate the transcription of those genes in peripheral tissues, and finally promote circadian oscillation [15–20]. Previous studies reported NPAS2 as a homolog of CLOCK; moreover, other vital clock homologous complexes such as CLOCK-BMAL1 or NPAS2-BMAL1 facilitate E-box-dependent transcription [21, 22]. NPAS2 is reported to compensate CLOCK [23–25]; therefore, any alterations in the form of deletions or mutations of NPAS2 could directly cause a complete disruption of the biological rhythmical order [26].

MicroRNAs (miRNAs) from a family of ~ 22 nucleotides in length and single-stranded non-coding RNA molecules are known to regulate gene expression at the post-transcriptional level by targeting their 3′ untranslated regions (3′UTRs) [27, 28]. Studies have confirmed that miRNAs play specific regulatory roles in the circadian rhythm. In mice, specific miRNAs such as miR-96, miR-124a, and miR-27b-3p have been found to oscillate in a circadian pattern [29, 30]. Other miRNAs such as miR-206 in mammalian skeletal muscle [31], miR-219, miR-132 and miR-142-3p in mice [32, 33], miR-263a, miR-263b and let-7 in *Drosophila* [34, 35], miR-182 in humans with depression [36], and miR-17-5p and miR-29b-3p in rats [37] have been widely reported.

Compared with other animals, birds have a more complex circadian system because their function requires pacemakers to be present in organs such as the pineal gland, retina, and SCN which regulates peripheral tissues [8, 38]. Oscillators in the pineal gland and SCN are known to be functionally involved in stabilizing and amplifying each other through their periodic release of secretions [39]. Reports suggest that numerous physiological outputs such as the daily egg-laying rhythm in birds are influenced by the coordination of circadian outputs through the various pacemakers present in the pineal gland and SCN [40, 41].

At the peak egg production period, chickens oviposit within 24 ~ 25 h cycles [42, 43], wherein a luteinizing hormone surge modulates the expression of genes related to the circadian clock [42]. Our previous study observed that the cosine expression of clock genes is involved in the regulation of the circadian clock in the uterus of chicken oviduct [43]. Other studies have reported the actions of specific clock miRNAs in mammals and *Drosophila*, however, there have been no reports in chickens. Therefore, in this study, we used RNA sequencing (RNA-seq) to identify clock-controlled miRNAs and explore their roles in signaling pathways in the chicken uterus.

## Results

### Morphological and histological characteristics of chicken uterus

**Fig. 1:**
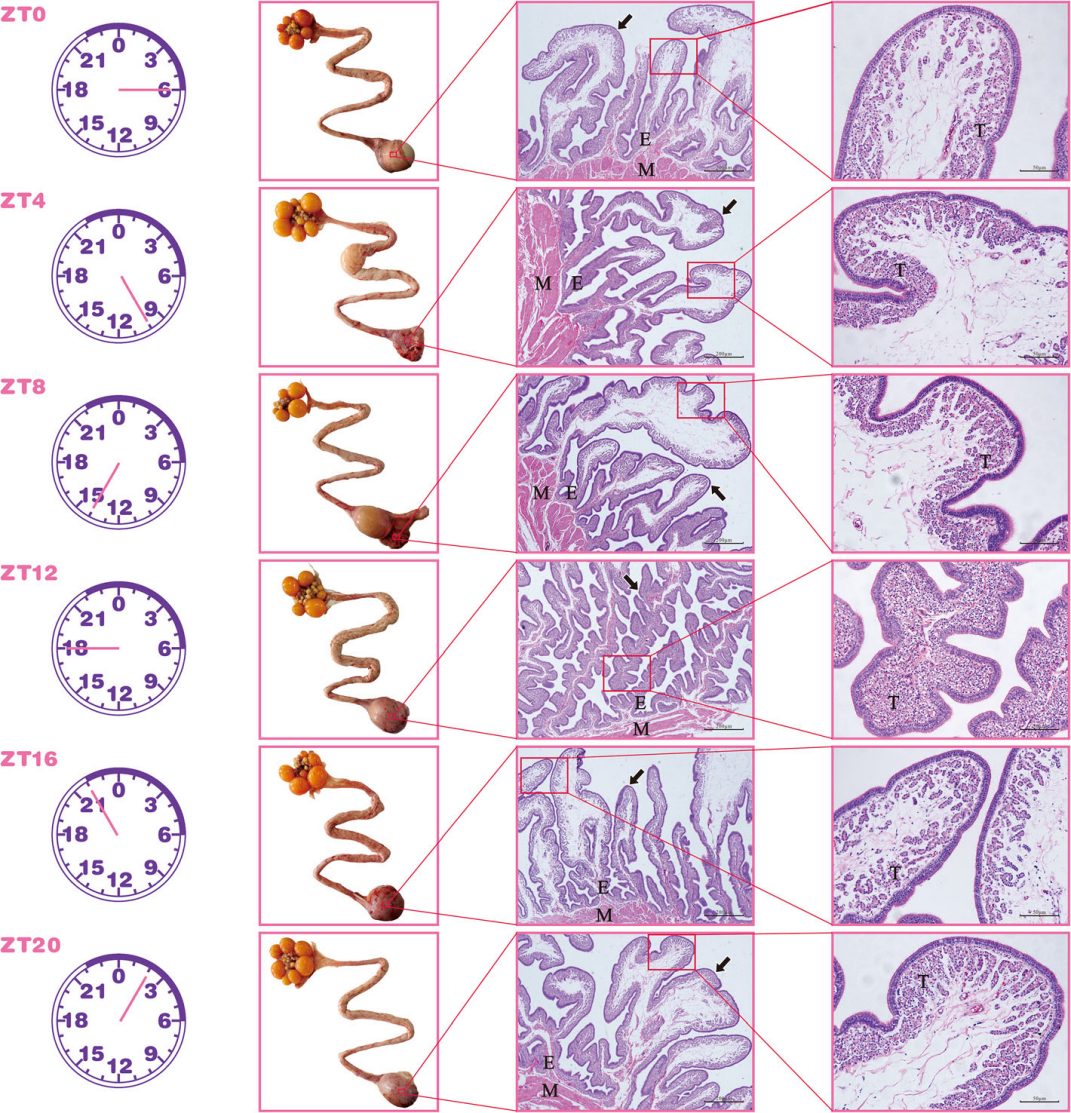
Chicken uterus morphology and histological characteristics. Hematoxylin-eosin (H&E) staining of chicken uterus at ZT4, ZT8, ZT12, ZT16, ZT20, and ZT0 (ZT24), respectively (200× magnification). Replications = 3. E: endometrium; M: myometrium; T: tubular gland cells. The black arrow indicates the endometrial glands

Zeitgeber time (ZT) is the nomenclature of time in the light-dark cycle. ZT0 (06:00 Beijing Time) was the time at which the lamps were turned on, and subsequent light simulation times were denoted as ZT4 (10:00 Beijing Time), ZT8 (14:00 Beijing Time), ZT12 (18:00 Beijing Time), ZT16 (22:00 Beijing Time), and ZT20 (02:00 Beijing Time), respectively. The uterus of 18 laying hens with similar ovipositional rhythmicity (each of three birds were sacrificed at 4 h intervals for 24 h) at ZT4, ZT8, ZT12, ZT16, ZT20, and ZT0 (ZT24) were collected. Results of the morphological or physical observations of the oviducts showed that eggs were present in the oviduct ampulla and isthmus at ZT4 and ZT8, respectively; whereas we also found that eggs were present in the uterus at ZT12, ZT16, ZT20 and ZT0. Based on histological observations, we found that the uterine glands in the endometrium had a folded and branched tubular structure. The density of the uterine glands increased gradually from ZT0 to ZT12 and decreased from ZT12 to ZT20. Importantly, at ZT12, the endometrium thickened, and both the length and folding of the uterine glands increased (Fig. 1). Moreover, the number of tubular gland cells increased and were neatly arranged; these secreted the uterine fluid containing various ions such as K^+^, Na^+^, HCO^3−^, and Ca^2+^ [44]. This may increase the contact area between the uterine tubular gland cells and the egg to rapidly secrete large amounts of uterine fluid.

### Identification of circadian miRNAs and miRNA-gene interaction network

**Fig. 2:**
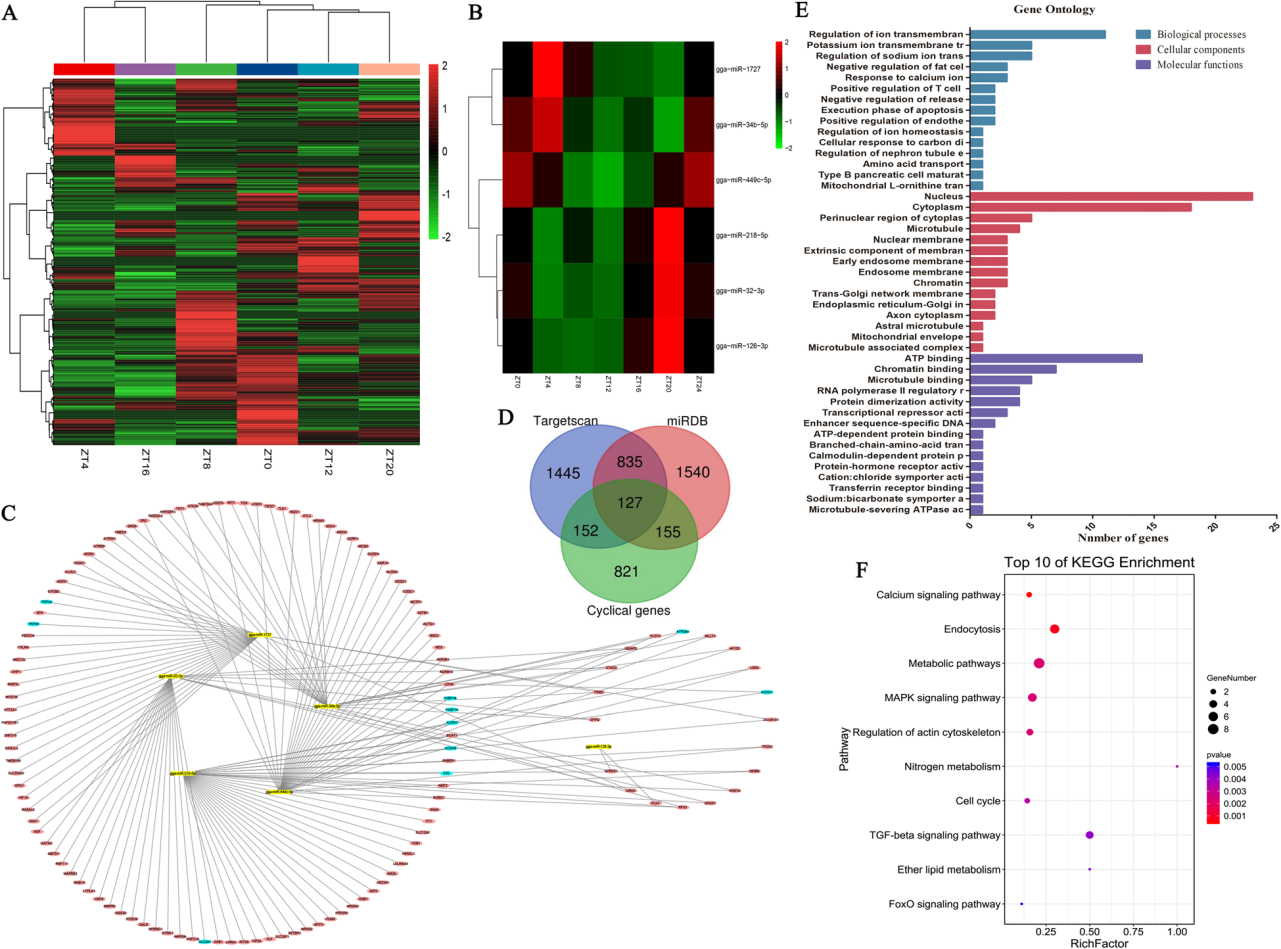
Identification of circadian miRNAs and functional analysis. **A** Heatmap of 734 miRNAs in chicken uterus at ZT4, ZT8, ZT12, ZT16, ZT20, and ZT0 (ZT24) time points. The value represents the log2 transformed values of (TPM + 1). **B** Heatmap of the six circadian miRNAs. **C** Six circadian miRNA-target gene correlation networks in the chicken uterus. Aqua green denotes ion transfer related genes. **D** Venn diagram showing the target genes of six circadian miRNAs using Targetscan and miRDB software, and uterine cyclical genes. **E** GO enrichment of the 127 circadian target genes. Top 15 terms of biological processes, cellular components, and molecular functions. The x-axis shows the number of genes. **F** KEGG [45] analysis showed the top 10 significantly enriched pathways associated with 127 circadian target genes

Transcriptome sequencing of chicken uterus at ZT4, ZT8, ZT12, ZT16, ZT20, and ZT0 (ZT24) was performed to identify the circadian miRNAs and to explore the regulatory mechanisms within the uterus of chickens. A total of 734 miRNAs were identified and expressed at least for one sampling time point and were included for further analysis of 18 small RNA libraries in the chicken uterus. The results obtained from Pearson’s correlation analysis (Supplementary Fig. 1) of the expression of miRNAs between samples showed that the correlation coefficient between sample biological repeats was high. Therefore, the average miRNA expression levels of the three biological replicates were used to plot the heatmap and showed as Fig. 2a. We combined JTK_CYCLE (v.3.4.3) and MetaCycle (v.1.2.0) (Lomb-Scargle and meta2d) algorithms to identify the circadian miRNAs, the details of which are shown in the Supplemental Material (circadian miRNA identification). In general, six miRNAs (gga-miR-218-5p, gga-miR-449c-5p, gga-miR-34b-5p, gga-miR-1727, gga-miR-32-3p, and gga-miR-126-3p) were identified as circadian miRNAs in the uterus (Fig. 2b), and the correlation network between 6 circadian miRNAs and their 127 circadian target genes were constructed (Fig. 2c). The results of the target genes of the 6 circadian miRNAs and uterine cyclical genes (our recently submitted uterus mRNA sequencing data) (PRJNA699682) are presented in a Venn diagram (Fig. 2d). Gene ontology (GO) and Kyoto Encyclopedia of Genes and Genomes (KEGG) pathway analyses were performed on 127 circadian target genes (Fig. 2e and f). The top 15 terms were involved in biological processes (BP) such as regulation of ion transmembrane transport, regulation of sodium ion transmembrane transporter activity, response to calcium ion, negative regulation of the release of sequestered calcium ions into the cytosol, cellular response to carbon dioxide, and positive regulation of endothelial cell migration (Fig. 2e). KEGG analysis showed that these circadian target genes were enriched in the calcium signaling pathway, endocytosis, metabolic pathways, MAPK signaling pathway, regulation of actin cytoskeleton, nitrogen metabolism, cell cycle, TGF-beta signaling pathway, ether lipid metabolism, and FoxO signaling pathway (Fig. 2f). The target genes of clock-controlled miRNAs involved in ion transfer during eggshell calcification are summarized in Table 1.

**Table 1:** Target genes of clock-controlled miRNAs involved in ion transfer during eggshell calcification

miRNAs	Target genes	Transfer type
gga-miR-218-5p	NPAS2, CA2 KCNH1/5/7	Catalyse HCO_3_^−^ formation (plasma membrane) Inward rectifiers K^+^ channels (plasma membrane)
gga-miR-449c-5p	ATP2B4 FKBP1A/B	Ca^2+^/H^+^ exchanger (plasma membrane) Ca^2+^ channel (endoplasmic membrane)
gga-miR-34b-5p	ATP2B4	Ca^2+^/H^+^ exchanger (plasma membrane)
gga-miR-1727	TRPV4/5	Ca^2+^ channel (plasma membrane)
gga-miR-32-3p	KCNH1 SLC4A7	Inward rectifiers K^+^ channels (plasma membrane) Na^+^/HCO_3_^−^ co-transporters (plasma membrane)
gga-miR-126-3p	SPRED1 PLK2	–

### Clock-controlled miR-449c-5p modulated ATP2B4 expression

**Fig. 3:**
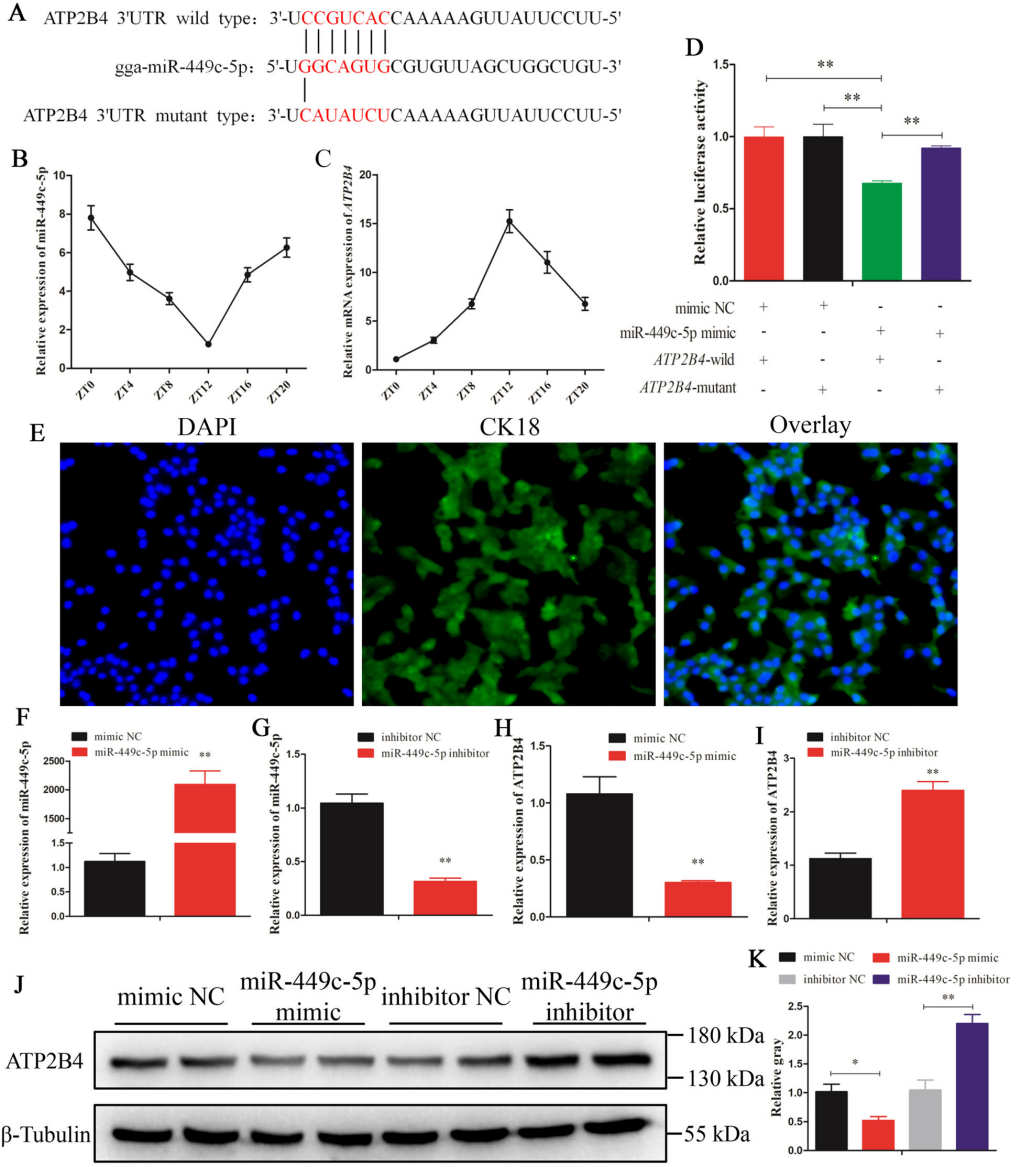
Clock-controlled miR-449c-5p inhibited ATP2B4 mRNA and protein expression by directly targeting ATP2B4 in uterine tubular gland cells. **A** The target position of the miR-449c-5p seed sequence on the ATP2B4–3′UTR sequence (red characters) was predicted using TargetScan software. **B**, **C** Relative expression of miR-449c-5p and ATP2B4 at the time point of ZT0 (ZT24), ZT4, ZT8, ZT12, ZT16, and ZT20 in the chicken uterus, respectively. **D** Chicken DF-1 cells were co-transfected with ATP2B4–3′UTR wild or mutant dual-luciferase vector and miR-449c-5p mimic or mimic-NC. Relative luciferase activity was assayed 48 h later. **E** Immunofluorescence analysis was performed to identify uterine tubular gland cells. **F**, **G** qRT-PCR was used to determine miR-449c-5p expression levels after transfection of miR-449c-5p overexpression and miR-449c-5p inhibition plasmid, respectively. **H**, **I** mRNA expression of ATP2B4 in chicken uterine tubular gland cells was detected by qRT-PCR after overexpression and inhibition of miR-449c-5p, respectively. **J**, **K** Protein expression of ATP2B4 in chicken uterine tubular gland cells was detected by Western blot analysis after a gain or loss of miR-449c-5p. UTR: untranslated region; miR: microRNA; DAPI: 4′, 6-diamidino-2-phenylindole; NC: negative control. Replications = 3. The samples were derived from the same experiment and the gels/blots were processed in parallel. The data are presented as mean ± standard error (SE); *P < 0.05 and **P < 0.01

We found that plasma membrane calcium-transporting ATPase 4 (ATP2B4) was predicted as the target gene of clock-controlled miR-449c-5p and was significantly enriched in the calcium signaling pathway. The results from Targetscan software prediction analysis showed that the seed region of miR-449c-5p was complementary to the 3′-UTR of the *ATP2B4* gene (Fig. 3a). Moreover, after determining the expression levels of miR-449c-5p and *ATP2B4* at different time points within a 24 h cycle in uterine tissues, we found a reduction in the expression level of miR-449c-5p from ZT0 to ZT12; however, it eventually increased from ZT12 to ZT20 (Fig. 3b). In contrast, the mRNA expression of *ATP2B4* increased, reached its highest level at ZT12, and then decreased sharply (Fig. 3c). Dual-luciferase reporter gene assay results showed that the luciferase activity of the ATP2B4 wild-type reporter vector decreased significantly in response to the miR-449c-5p mimic while no dramatic changes were observed in the mutant vector (Fig. 3d), indicating that *ATP2B4* is a target gene of miR-449c-5p.

To investigate the function of miR-449c-5p in Ca^2+^ transfer in the uterus, immunofluorescence analysis was performed to identify the uterine tubular gland cells. Cytokeratin 18 (CK18) is a specific cytokeratin uterine tubular gland cell marker [46, 47]. Immunofluorescence analysis showed that the chicken uterine tubular gland cells were isolated and cultured (Fig. 3e). miR-449c-5p expression increased significantly after transfection with the miR-449c-5p mimic (*P* < 0.05) (Fig. 3f), but its expression decreased after transfection with miR-449c-5p inhibitor (Fig. 3g).

**Fig. 4:**
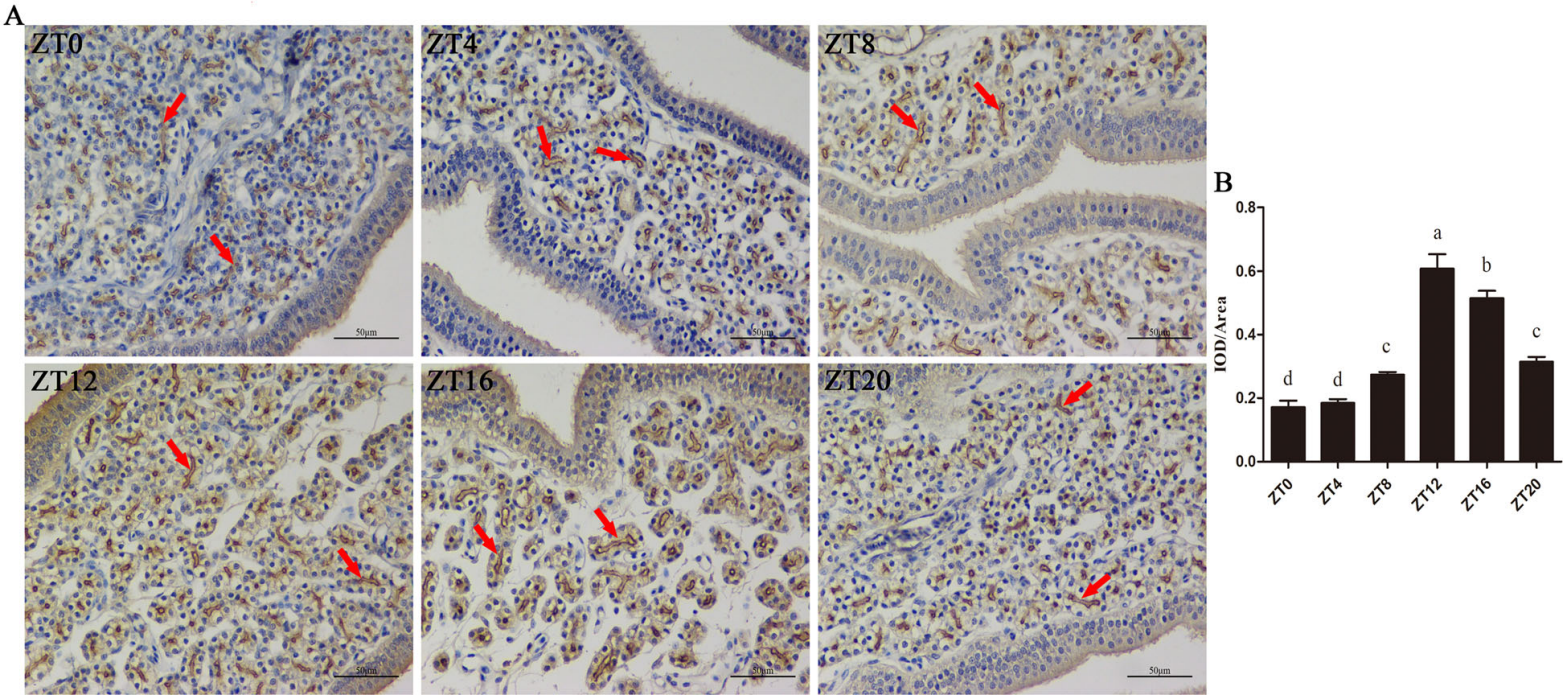
ATP2B4 immunohistochemistry in chicken uterus. **A** Immunohistochemical staining with the ATP2B4 antibody visualized using chromogen diaminobenzene (brown staining) in the chicken uterus. The arrows indicate the relative areas of positive staining. **B** Digital conversion histogram; each point represents the mean ± SE. Different lowercase letters indicate significant differences among the groups (*P* < 0.05)

The mRNA and protein levels of ATP2B4 decreased significantly due to the overexpression of miR-449c-5p (Fig. 3h, j, and k), whereas the results obtained after the inhibition of miR-449c-5p showed that the mRNA and protein expression of ATP2B4 increased significantly (Fig. 3i, j, and k). Immunohistochemistry showed that the protein expression of ATP2B4 in the uterus increased initially and then gradually decreased from ZT0 to ZT20 with the highest expression at ZT12 (Fig. 4a and b), which was similar to the trend of the mRNA expression of *ATP2B4*.

### ATP2B4 regulated Ca^2+^ transfer in uterine tubular gland cells

**Fig. 5:**
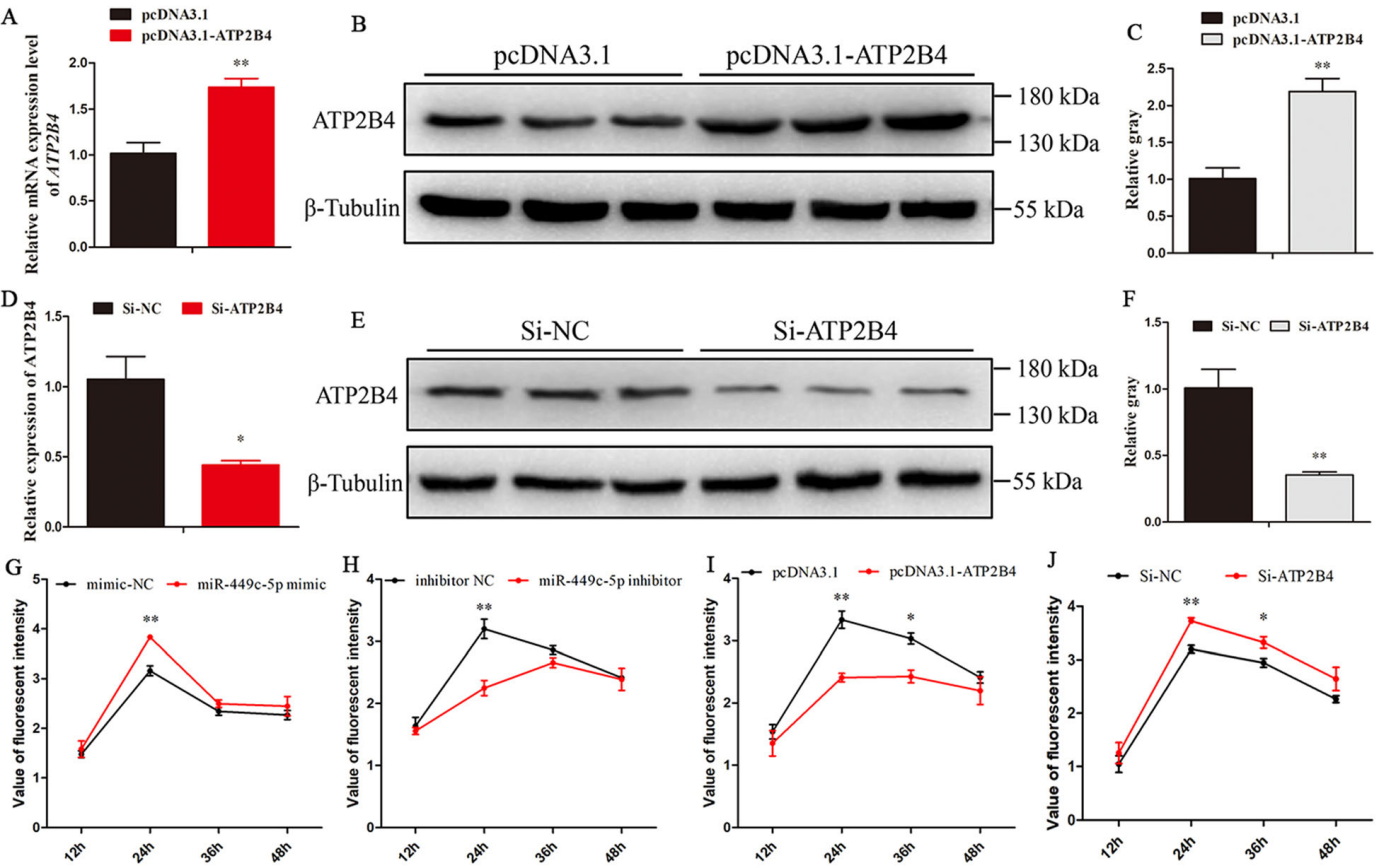
ATP2B4 regulated Ca^2+^ transfer in the uterine tubular gland cells. **A**, **B** and **C** mRNA and protein expression of ATP2B4 was detected after transfection of the overexpression plasmid (pcDNA3.1-ATP2B4) and empty pcDNA3.1 vector. **D**, **E**, and **F** mRNA and protein expression of ATP2B4 was detected after transfection with small interfering RNA (Si-ATP2B4) and siRNA negative control (Si-NC). **G** and **H** The fluorescence intensity was measured using a microplate reader indicating the intracellular concentration of Ca^2+^ after overexpression and inhibition of miR-449c-5p. **I** and **J** Intracellular concentrations of Ca^2+^ ions were detected after transfection overexpression and inhibition of ATP2B4. Replications = 3. The samples were derived from the same experiment and the gels/blots were processed in parallel. Data are presented as mean ± standard error (SE); *P < 0.05 and **P < 0.01

**Fig. 6:**
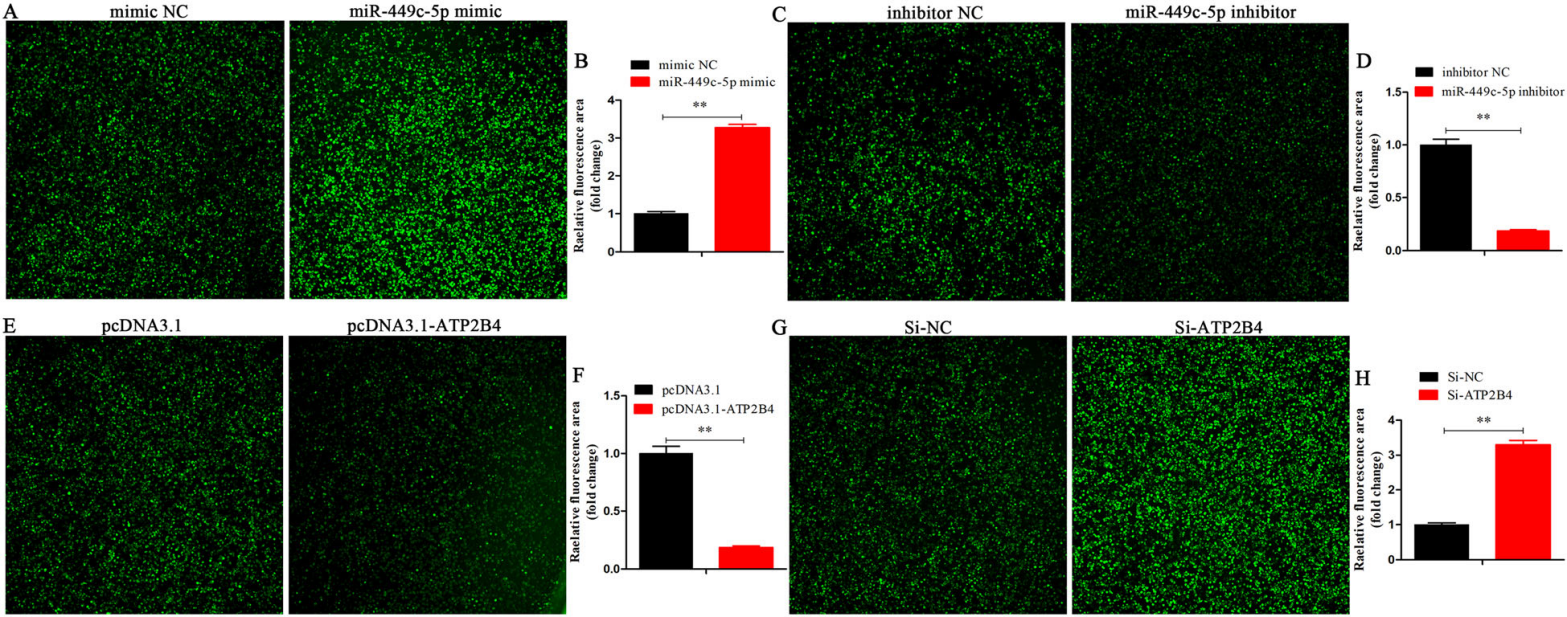
Clock-controlled miR-449c-5p regulates Ca^2+^ transport by targeting ATP2B4 in chicken uterine tubular gland cells. **A** and **B** Fluorescence intensity was observed and analyzed after overexpression of miR-449c-5p. **C** and **D** Fluorescence intensity was observed and analyzed after inhibition of miR-449c-5p. **E**, **F**, **G**, and **H** Fluorescence intensity was observed and analyzed after transfection, overexpression, and inhibition of ATP2B4. Replications = 3. Data are presented as mean ± standard error (SE); *P < 0.05 and **P < 0.01

The primary uterine tubular gland cells of chicken were isolated and cultured. The mRNA and protein abundances of ATP2B were determined after transfection with pcDNA3.1- ATP2B4 and pcDNA3.1 empty plasmid, or Si-ATP2B4 and Si-NC. Compared with the pcDNA3.1, the group pcDNA3.1-ATP2B4 significantly increased mRNA and protein levels of ATP2B4 (Fig. 5a, b, and c). Meanwhile, the group Si-ATP2B4 had lower mRNA and protein levels of ATP2B4 than the group Si-NC (Fig. 5d, e, and f). Compared with the control group (mimic NC), overexpression of miR-449c-5p significantly increased the fluorescence value after transfection for 24 h, indicating an increase in intracellular Ca^2+^ concentration (Fig. 5g). Knockdown of miR-449c-5p reduced the concentration of Ca^2+^ (Fig. 5h). Moreover, *ATP2B4* overexpression significantly decreased the concentration of Ca^2+^ after transfection for 24 h and 36 h (Fig. 5i). These results are similar to the down-regulation of miR-449c-5p, but contrary to the knockdown of *ATP2B4* (Fig. 5j). Moreover, a BBcellProbe F03 fluorescence probe was used to measure the calcium ion concentration in the uterine tubular gland cells, which was combined with the intracellular calcium ions to produce strong fluorescence. The results showed that the fluorescence intensity was significantly higher in miR-449c-5p overexpression (Fig. 6a and b) and Si-ATP2B4 (Fig. 6g and h) groups than in the mimic NC and Si-NC groups, respectively. However, there was decreased fluorescence intensity in the miR-449c-5p knockdown (Fig. 6c and d) and *ATP2B4* overexpression groups (Fig. 6e and f). All results indicated that ATP2B4 regulates uterine Ca^2+^ trans-epithelial transport.

## Discussion

The vital internal devices that run on an approximate 24 h cycle and respond to external rhythms through phase resetting are considered to be circadian clocks. Almost all living organisms possess circadian timekeeping mechanisms that help monitor and regulate daily rhythms of physiological and behavioral activities [42, 43].

Chickens oviposit within a 24 ~ 25 h rhythm during peak egg laying periods. At this stage, clock genes in the oviduct exhibit cosine expression patterns [42, 43], indicating that the circadian clock plays a vital regulatory role in the chicken uterus. Cheng et al. reported that miRNAs regulate the circadian clock [32]. Therefore, to better understand the roles played by specific miRNAs in eggshell calcification in the chicken uterus, we used transcriptome sequencing to explore clock-controlled miRNA functions and their regulatory effects. The results revealed six special clock-controlled miRNAs and their related pathways that play critical roles in eggshell calcification in the chicken uterus.

Shell calcification during egg formation requires the continuous supply of large amounts of calcium and carbonate ions from the uterine fluid, which are derived from the blood stream via trans-epithelial transport across the uterine gland cells [48, 49]. The developing egg is observed to inflate and rotate in the uterus during the rapid phase of shell calcification (between 10 and 22 h postovulation) [50]. In the present study, histological characteristics of the uterus showed that the number of tubular gland cells increased, and they were neatly arranged at ZT12, which has been reported as the time point for secretion of uterine fluid including various ions needed for eggshell calcification [44]. From the results, we ddeduce that ZT12 is the rapid phase of egg shell calcification in chickens and the rapid phase lasts 8 h (starting at time point ZT8 and ending before ZT16).

**Fig. 7:**
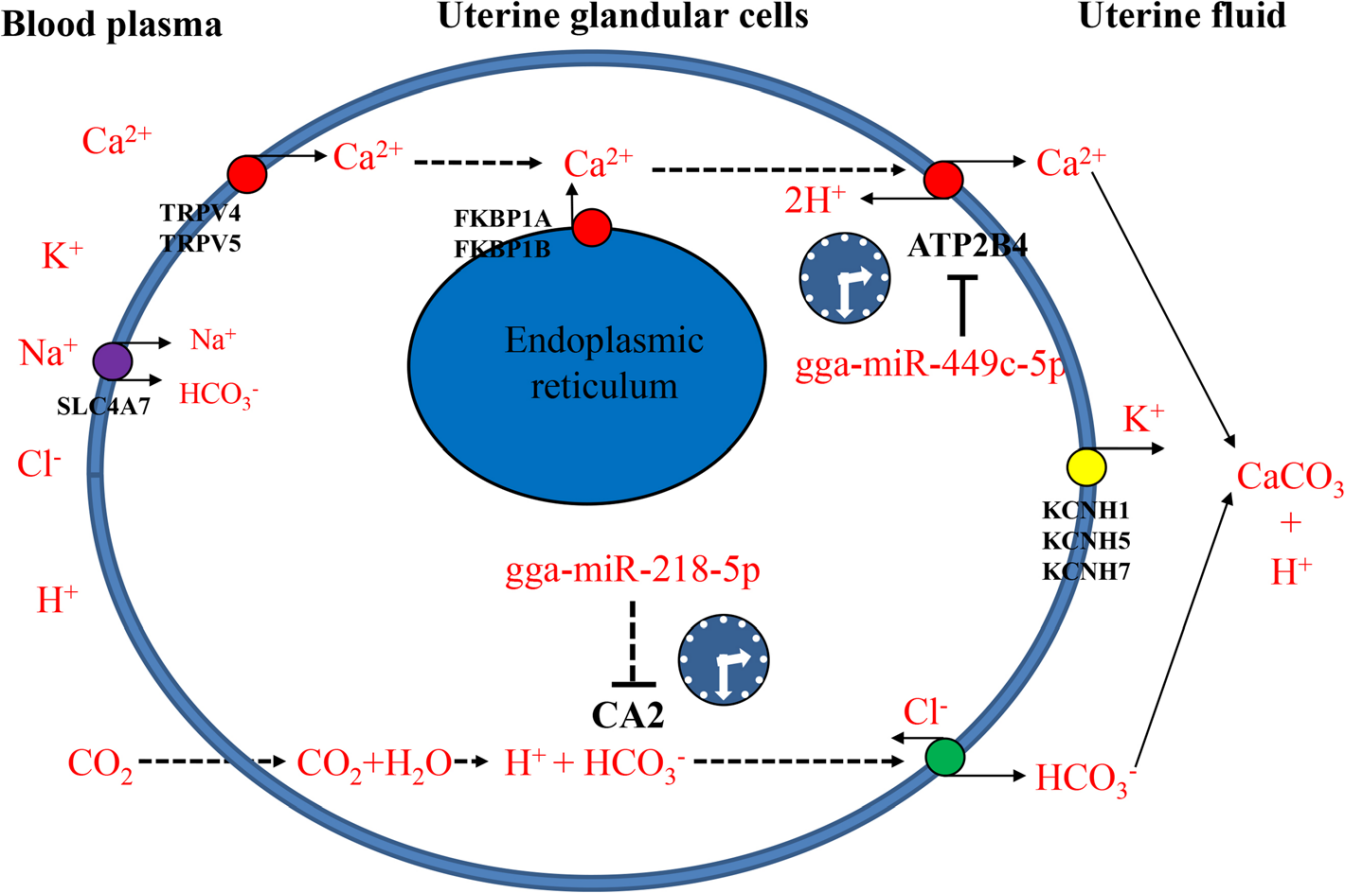
The general model describing clock-controlled miRNA regulates ion transporters during eggshell calcification in the chicken uterus. This figure summarizes the general mechanisms involved in the regulation of the transport, distribution, and transformation of Ca^2+^ from the blood plasma through the uterine tubular gland cell membrane (Ca^2+^ trans-epithelial transport) and then suspended in the uterine fluid in a usable form (CaCO_3_) for utilization in eggshell calcification. Clock-controlled miR-449c-5p in the uterus of chickens regulates Ca^2+^ transport by targeting ATP2B4 during eggshell calcification. ATP2B4 was responsible for utilizing the stored energy in the form of ATP to extrude Ca^2+^ from the cell against the electrochemical gradient and it was also involved in the active transport of calcium out of the tubular gland cells into the calcium-rich fluid of the uterine lumen. *NPAS2*, one of the core clock genes, was predicted to be the target gene of clock-controlled miR-218-5p, and another target gene *CA2* was related to the carbonic anhydrase activity of the hen oviduct

Dysregulation or dysfunction of miRNA(s) results in a total reduction in cellular functions. For instance, miR-449c-5p has been reported to suppress osteogenic differentiation of valve interstitial cells [51] . Hence, miR-449c-5p could be a potential target for treating calcific aortic valve disease. In this experiment, our results indicated that the levels of miR-449c-5p in the chicken uterus showed a pattern of cosine expression in the verification experiment. This indicated that a biological clock regulates miR-449c-5p. GO and KEGG results showed that the target genes of these six circadian miRNAs were mainly associated with biological processes including the regulation of ion transmembrane transport, response to calcium ion, and calcium signaling pathway enrichment. Importantly, plasma membrane Ca^2+^-transporting ATPase 4 (ATP2B4) is a predicted target gene of clock-controlled miR-449c-5p and is highly enriched in the calcium signaling pathway (Fig. 7). A previous study localized ATP2B4 in uterine tubular gland cells [52], and reported the promotion of trans-epithelial transfer of Ca^2+^ into the uterine fluid in avian species [49]. We deduced that clock-controlled miR-449c-5p may regulate Ca^2+^ transport during eggshell calcification in the chicken uterus.

In the cellular system, Ca^2+^ is regarded as one of the most important ions because of its active involvement in cellular excitation and also serves as a vital second messenger. Hence, maintaining this electrochemical gradient is critical for normal cell physiological functioning and this requires an energy dependent mechanism of Ca^2+^ expulsion or conversion into a stable form (CaCO_3_) [53]. In chickens, eggshell formation takes place daily in the uterus of the oviduct and is one of the most rapid mineralization processes or physiological phenomena known [50]; during this process, large amounts of calcium carbonate (CaCO_3_) are required. Neither of the involved elements (Ca^2+^ and HCO_3_^−^) are stored in the uterus but are continuously supplied during eggshell formation by the blood plasma via trans-epithelial transport which takes place across the uterine glandular cells [52, 54–58].

Plasma membrane calcium ATPases (ATP2Bs) is the main regulator of intracellular Ca^2+^ levels. Ca^2+^ secretion from tubular gland cells is transported into the uterine fluid to actively form a part of the Ca^2+^-ATPase [56, 58]. Plasma membrane Ca^2+^ ATPases are ubiquitously expressed in the plasma membrane and use ATP in the form of energy to pump Ca^2+^ out of the cells. In general, four paralogs ATP2B1, ATP2B2, ATP2B3, and ATP2B4 are found in mammalian cells but only three (ATP2B1, B2, B4) are conserved in birds [59]. These proteins are similar, but differ in their tissue expression and activation speed. The last step of uterine Ca^2+^ trans-epithelial transport is the output from the glandular cells, which occurs against a concentration gradient. Therefore, Ca^2+^ secretion in the uterine fluid occurs via an active process, involving Ca^2+^-ATPase [58, 60, 61]. Plasma membrane calcium-transporting ATPase 4 (ATP2B4) is a subunit of plasma membrane Ca^2+^-ATPase isoform 4 (PMCA4) [62]. The activities and expression of Ca^2+^-ATPase are associated with the periods of eggshell calcification especially when high concentrations of calcium ions are required for eggshell formation [58]. In the current study, we found that there was a rhythmic expression of the mRNA and protein levels of ATP2B4 and our confirmatory experiment confirmed that miR-449c-5p directly targeted ATP2B4. The results indicated that the expression of miR-449c-5p was opposite to that of ATP2B4 within a 24 h cycle in the chicken uterus, and it was also revealed that miR-449c-5p inhibited mRNA and protein expression of ATP2B4 in uterine tubular gland cells.

Previous reports describe ATP2B4 as the main mechanism found in the eggshell gland (ESG) of laying birds and is responsible for utilizing stored energy in the form of ATP to extrude Ca^2+^ from the cell against the electrochemical gradient [63, 64]. Another study identified and localized ATP2B4 in the uterine tubular gland cells of King Quail and confirmed its involvement in the active transport of calcium out of the tubular gland cells into the calcium-rich fluid of the uterine lumen [52]. In this study, we further explored the role of ATP2B4 in the transmembrane transport of calcium ions in the uterine tubular gland cells and found that *ATP2B4* overexpression significantly decreased the intracellular Ca^2+^ concentration but significantly increased with the transfection of *ATP2B4* knockdown, indicating that ATP2B4 promotes uterine Ca^2+^ trans-epithelial transport. Furthermore, miR-449c-5p showed similar changes in Ca^2+^ concentration upon knockdown of *ATP2B4*, which was contrary to *ATP2B4* overexpression.

## Conclusions

In conclusion, we identified six circadian miRNAs in the chicken uterus within a 24 h cycle. GO and KEGG analyses of 127 circadian target genes showed that the target genes were mainly associated with biological processes including; the regulation of ion transmembrane transport, response to calcium ion, and enrichment of the calcium signaling pathway. Our results indicated that circadian miR-449c-5p in the chicken uterus showed a pattern of cosine expression in the verification experiment. *ATP2B4* is the target gene of clock-controlled miR-449c-5p and is highly enriched in the calcium signaling pathway. Subsequently, we detected that clock-controlled miR-449c-5p in chicken uterus regulated Ca^2+^ transport by targeting *ATP2B4* during eggshell calcification (Fig. 7).

## Methods

### Animals

A total of 500 30-week-old laying hens (Line BH-01, bred by Sichuan Agriculture University for six generations with black shanks and dotted yellow feathers) were raised under a photoperiod of 16 h of light and 8 h of darkness (16 L: 8D). Their oviposition time was monitored and recorded every 30 min from 06:00 Beijing Time to 16:00 Beijing Time by artificial observation. The light in the chicken’s pen was turned on at 06:00 Beijing Time and turned off at 20:00 Beijing Time. Illumination was provided by one row of un-shaded incandescent lamps (25 W); the mean luminance at a height of 2 m was 15 Lux.

### Sample collection and RNA extraction

Eighteen hens with similar oviposition times were sacrificed at ZT4, ZT8, ZT12, ZT16, ZT20, and ZT0 (ZT24) (each of three birds at successive 4-h intervals) by cervical dislocation and their uterine tissues were collected. All uterine samples were quickly frozen in liquid nitrogen and stored at − 80 °C until assayed for RNA and qRT-PCR analyses.

### Morphological observation and histological staining

The uterine tissues were cut into sections and embedded in paraffin for 24 h to observe the morphological changes. Thereafter, sections were stained with hematoxylin and eosin (H&E) for observation under a fluorescence microscope (DP80; Olympus, Japan); and 10 fields were randomly selected for statistical analysis.

### Library construction and RNA-Seq

Total RNA was isolated from uterine tissues using TRIzol Reagent (Invitrogen, CA, USA) following the manufacturer’s protocol. We determined the concentration and purity of RNA samples, and the integrity of 18S and 28S rRNA bands using the A260/280 absorbance ratio and 2% agarose gel electrophoresis respectively. The cDNA libraries of small RNAs were generated using a Truseq™ RNA sample prep kit (Illumina) according to the manufacturer’s instructions and RNA sequencing was performed using an Illumina Hiseq 2500 system (Denovo Gene, Guangzhou, China).

### Bioinformatics analysis

In this study, we filtered the raw reads to obtain clean reads as previously described [65]. Briefly, clean reads were obtained by removing reads containing ploy-N, with 5′ adapter contaminants, without 3′ adapter or the insert tag, containing ploy A or T or G or C, shorter than 18 nt (adapters were not included) and low-quality reads from raw data (Supplementary Table 1). To remove tRNA, snRNA, snoRNA, rRNA, fragments from mRNA degradation, and repeat sequences, the clean reads were aligned with the GenBank database (Release 209.0), the Rfam database (11.0), and the reference genome. For the remaining reads, miRBase 21.0 was used to search and identify known miRNAs in other species and known miRNAs in chicken. miRNA expression levels were calculated and normalized to the transcript per million (TPM). The average miRNA expression levels of the three independent biological replicates were used to plot the clustering heatmaps. The clustering algorithm was used in the ward. D and distance were measured using Euclidean distance. The expressed miRNAs were calculated and plotted in heatmaps (R software v.3.2.4.).

### Identification of circadian miRNAs

The JTK_CYCLE (v.3.4.3) [66] and MetaCycle (v.1.2.0) (Lomb-Scargle and meta2d) algorithms [67] were used to analyze periodic data. Our samples were uterine tissues of laying hens at 6 time points, and there were 3 biological duplicates at each time point. JTK_CYCLE can improve power in analyzing datasets with duplicate samples and Lomb-Scargle has a good classifier of periodic signals and noise. Therefore, we combined JTK_CYCLE and Lomb-Scargle to identify the circadian miRNAs, and the results were represented as adjusted *P*-value and PER period values. The *P* values denote the significance of miRNA rhythmic expression, whereas the PER value represents the rhythm cycle time. miRNAs with adjusted *P*-values < 0.05 and a periodic PER value of 20–24 were considered candidate circadian miRNAs. Thereafter, we constructed a regulatory interaction network between clock-controlled miRNAs and their target genes using integrative miRNA target-prediction Targetscan (http://www.targetscan.org/vert_72/) and miRDB (http://mirdb.org/index.html) [68] and network-analysis (Cytoscape software v.3.7.1) [69]. We further conducted GO and KEGG pathway enrichment analyses to identify the biological functions of the target genes using DAVID and KOBAS v.2.0 software respectively. The results with a *P*-value < 0.05 were considered to be significantly enriched.

### Dual-luciferase reporter assay

The chicken embryo fibroblast cell line (DF-1) was seeded in 48-well cell plates and cultured in growth medium containing F12 (Hyclone, State of Utah, USA) and 10% fetal bovine serum (Gibco, Langley, OK) in a cell culture incubator at 37 °C, 5% CO_2_ and 95% air saturated humidity. After reaching a cell density coverage of 70 ~ 80%, the plasmid (ATP2B4–3′UTR wild type or mutant type) was co-transfected with mimic negative control (NC) and miR-449c-5p respectively. After 48 h luciferase activity was tested using a luciferase reporter assay kit (Promega, Madison, WI, USA) following the manufacturer’s instructions.

### Immunohistochemical analysis

Uterine samples were collected at these time points ZT4, ZT8, ZT12, ZT16, ZT20, and ZT24, and were washed in sterile PBS three times. Thereafter, they were fixed in 4% paraformaldehyde at room temperature (RT) for 20 min, after which they were treated with hydrogen peroxide solution (3%) to deactivate the endogenous enzymes. Subsequently, the samples were washed in PBS solution for 5 min, and then a blocking reagent (goat serum) was added at RT for 20 min after which they were incubated with primary antibody rabbit anti-ATP2B4 (Abcam, Cambridge, UK) overnight at 4 °C. After incubation, the samples were washed and incubated with fluorescence-labeled secondary antibody at RT for 30 min. After the second incubation, the samples were further washed in PBS and incubated for the third time with peroxidase (POD)-labeled streptavidin (DyLight 488) at RT for 30 min. A DAB kit (BBI, Canada) was used for color development at RT for 5 ~ 30 min, which was followed by observation, and photomicrographs were obtained using a light microscope (Nikon Eclipse E100, Japan) equipped with an imaging system (Nikon DS-U3, Japan). The images obtained were analyzed using Image-Pro Plus software (version 6.0, Media Cybernetics, Silver Spring, USA).

### Uterine tubular gland cell culture and transfection

Both ends of the uterine tissue were ligated with a cotton thread and were repeatedly dissected and cleaned with sterile Hank’s balanced salt solution; thereafter, the endometrial tissue was collected and cut into pieces. The cells were digested with collagenase (1 mg/mL; type I, Sigma) in a water bath at 37 °C for 50 ~ 60 min, and then centrifuged, after which the supernatant was discarded. The cells were resuspended in a growth medium containing F12 (Hyclone) + 10% fetal bovine serum (Gibco) + 0.1% penicillin/streptomycin (Invitrogen, Carlsbad, CA, USA), and were seeded in 75 cm^2^ cell culture bottles (T75) (Costar, Cambridge, MA, USA). They were then cultured in a cell culture incubator at 37 °C, 5% CO_2_, and 95% air saturated humidity for 3 h before the supernatant was filtered (using cell sieve No.200). Cell counts were performed before they were placed in a 6-well plate (1 × 10^6^ cells/well) for further culturing [70]. Cell transfection was performed after the cells reached a coverage density of 70–80% using lipofectamine 3000 reagent (Invitrogen, USA), according to the manufacturer’s instructions. The miR-449c-5p mimic, miR-449c-5p inhibitor, mimic negative control (mimic NC), inhibitor NC, small interfering RNA (Si-ATP2B4), siRNA negative control (Si-NC), ATP2B4 overexpression plasmid (pcDNA3.1-ATP2B4), and empty pcDNA3.1 vector used in this study were designed and purchased from RiboBio (Guangzhou, China).

### Immunofluorescence analysis

Immunofluorescence analysis was performed to identify the tubular gland cells of the chicken uterus. Uterine tubular gland cells were placed in a 6-well plate, and washed with PBS for 5 min. Subsequently, the cells were fixed in 4% paraformaldehyde for 10 min and washed again, and 0.2% Triton X-100 was added to ensure permeability of the cell membrane for 10 min. The cells were washed and subsequently incubated overnight at 4 °C with rabbit anti-Cytokeratin 18 (Bioss, Beijing, China). The next morning, the cells were washed and incubated with fluorescence-labeled secondary antibodies at room temperature for 1 h. Cells were finally washed in Tris-Buffered Saline Tween-20 (TBST) and florescence intensity was observed and analyzed using a fluorescence microscope (DP80; Olympus, Japan).

### Calcium ion detection in uterine tubular gland cells

The cells were cultured in 96-well plates, and then a calcium ion detection kit (BBcellProbe F03, BestBio Biotech Co. Ltd., Shanghai, China) was used to measure the calcium ion concentration in uterine tubular gland cells following the manufacturer’s instructions. The BBcellProbe F03 fluorescence probe was combined with intracellular calcium ions to produce strong fluorescence. Fluorescence intensity was measured using a microplate reader (Thermo Fisher, Varioskan LUX, USA) at an excitation wavelength of 490 nm and an emission wavelength of 516 nm. Subsequently, the fields were observed and photographed using a fluorescence microscope (DP80; Olympus, Japan). Three fields were randomly selected and Image-Pro plus software was used for statistical analysis.

### Quantitative real-time PCR (qRT-PCR)

qRT-PCR analysis was conducted with a reaction volume of 10 μL containing 5 μL TB GreenTM Premix (Takara), 0.5 μL forward and reverse primers, 1 μL cDNA, and 3 μL DNase/RNase-Free Deionized Water (Tiangen, Beijing, China). The reaction conditions followed the protocols and instructions. Chicken GAPDH and U6 were used as the internal controls. According to a gene bank, the primers were designed by Oligo 6.0 software and Primer premier 5.0 software; the primers used are listed in Table 2.

**Table 2:** Primers used for qRT-PCR

Gene	Sequence (5′ - 3′)	Product Length (bp)	Annealing Temperature (°C)
*ATP2B4*	F: CCTCCGTCAATTCCACTCCC	89	58
	R: CTACGGAACGCATTCACCAC		
*GAPDH*	F: TCCTCCACCTTTGATGCG	146	59
	R: GTGCCTGGCTCACTCCTT		

*F* Forward primer, *R* Reverse primer

### Western blotting assay

Uterine tubular gland cells were lysed in lysis buffer (BestBio) and the total protein concentration was quantified using a BCA assay (BestBio) according to the manufacturer’s protocol. Immunoblots were performed using primary and secondary antibodies such as anti-ATP2B4 (PMCA4) (1:1000, Abcam) and goat anti-mouse IgG (Zen-Bio, Chengdu, China) respectively. Western blotting was performed as described previously [71].

### Statistical analysis

Data are expressed as the mean ± standard error (SE). Statistical significance was assessed using one-way ANOVA followed by Duncan’s multiple range test. SAS 9.3 (SAS Inst., Cary, North Carolina, USA) was used for all statistical analyses and GraphPad Prism Software 5.01 (Graph Pad Inc., La Jolla, CA) was used for the imaging. Differences were considered significant at *P* < 0.05 (*) and *P* < 0.01 (**).

## Supplementary Information

**Additional file 1.**

**Additional file 2.**

## Data Availability

The data used to support the findings of this study are available from the corresponding author upon request. The raw data has been submitted to the National Center for Biotechnology Information (NCBI) Sequence Read Archive (SRA, https://submit.ncbi.nlm.nih.gov/subs/sra/); accession number (PRJNA698298).

## Abbreviations

ATP2B4: Ca^2+^-transporting ATPase 4
SCN: Suprachiasmatic nucleus
3′ UTRs: 3′ untranslated regions
miRNAs: MicroRNAs
RNA-seq: RNA sequencing
ZT: Zeitgeber time
GO: Gene ontology
KEGG: Kyoto Encyclopedia of Genes and Genomes
DF-1: Chicken embryo fibroblast cell line
RT: Room temperature
TBST: Tris-Buffered Saline Tween-20
CK18: Cytokertin 18
PMCA4: Plasma membrane Ca^2+^-ATPase isoform 4
ESG: Eggshell gland
