# Supplementary material for: Circadian miR-449c-5p regulates uterine Ca2+ transport during eggshell calcification in chickens
Source: BMC Genomics. 2021 Oct 26;22:764. doi: 10.1186/s12864-021-08074-3 (PMC8547053; doi:10.1186/s12864-021-08074-3)
Supplement: Supplementary file 1 — Additional file 1. [file 12864_2021_8074_MOESM1_ESM.docx]

Supplementary Table 1

Table S1 Data filtering

| Sample | Total reads | N% > 10% | Low quality | 5 adapter  contamine | 3 adapter null or insert  null | with  ployA/T/G/C | Clean reads |
| --- | --- | --- | --- | --- | --- | --- | --- |
| ZT4_1 | 14957851  (100.00%) | 1626  (0.01%) | 15234  (0.10%) | 3359 (0.02%) | 987244 (6.60%) | 26034 (0.17%) | 13924354  (93.09%) |
| ZT4_2 | 15147826  (100.00%) | 1656  (0.01%) | 15442  (0.10%) | 399 (0.00%) | 589864 (3.89%) | 4608 (0.03%) | 14535857  (95.96%) |
| ZT4_3 | 14261873  (100.00%) | 283 (0.00%) | 21425  (0.15%) | 853 (0.01%) | 457233 (3.21%) | 16877 (0.12%) | 13765202  (96.52%) |
| ZT8_1 | 12714123  (100.00%) | 1415  (0.01%) | 14773  (0.12%) | 711 (0.01%) | 475939 (3.74%) | 15337 (0.12%) | 12205948  (96.00%) |
| ZT8_2 | 11492476  (100.00%) | 1417  (0.01%) | 25262  (0.22%) | 789 (0.01%) | 385873 (3.36%) | 15398 (0.13%) | 11063737  (96.27%) |
| ZT8_3 | 12123685  (100.00%) | 1438  (0.01%) | 27289  (0.23%) | 545 (0.00%) | 374768 (3.09%) | 11765 (0.10%) | 11707880  (96.57%) |
| ZT12_1 | 13125093  (100.00%) | 292 (0.00%) | 40719  (0.31%) | 876 (0.01%) | 488058 (3.72%) | 14438 (0.11%) | 12580710  (95.85%) |
| ZT12_2 | 14021208  (100.00%) | 352 (0.00%) | 56424  (0.40%) | 1244 (0.01%) | 420752 (3.00%) | 24027 (0.17%) | 13518409  (96.41%) |
| ZT12_3 | 15313658  (100.00%) | 391 (0.00%) | 56422  (0.37%) | 1567 (0.01%) | 549359 (3.59%) | 23946 (0.16%) | 14681973  (95.88%) |
| ZT16_1 | 14833047  (100.00%) | 1777  (0.01%) | 32210  (0.22%) | 2142 (0.01%) | 502307 (3.39%) | 25375 (0.17%) | 14269236  (96.20%) |
| ZT16_2 | 11594160  (100.00%) | 1467  (0.01%) | 21039  (0.18%) | 507 (0.00%) | 402997 (3.48%) | 7360 (0.06%) | 11160790  (96.26%) |
| ZT16_3 | 10813216  (100.00%) | 1337  (0.01%) | 22070  (0.20%) | 787 (0.01%) | 376562 (3.48%) | 16825 (0.16%) | 10395635  (96.14%) |
| ZT20_1 | 13279451  (100.00%) | 1615  (0.01%) | 29170  (0.22%) | 796 (0.01%) | 150131 (1.13%) | 15265 (0.11%) | 13082474  (98.52%) |
| ZT20_2 | 11857874  (100.00%) | 1454  (0.01%) | 23343  (0.20%) | 570 (0.00%) | 133132 (1.12%) | 9505 (0.08%) | 11689870  (98.58%) |
| ZT20_3 | 13261883  (100.00%) | 1510  (0.01%) | 29612  (0.22%) | 1170 (0.01%) | 172204 (1.30%) | 17584 (0.13%) | 13039803  (98.33%) |
| ZT24_1 | 13953299  (100.00%) | 311 (0.00%) | 59574  (0.43%) | 669 (0.00%) | 142268 (1.02%) | 14815 (0.11%) | 13735662  (98.44%) |
| ZT24_2 | 14221470  (100.00%) | 320 (0.00%) | 57674  (0.41%) | 386 (0.00%) | 140304 (0.99%) | 11221 (0.08%) | 14011565  (98.52%) |
| ZT24_3 | 13889204  (100.00%) | 315 (0.00%) | 60448  (0.44%) | 867 (0.01%) | 132650 (0.96%) | 21642 (0.16%) | 13673282  (98.45%) |

N: the base information cannot be determined; low-quality reads: the number of bases with mass Q-value ≤ 5 accounts for more than 50% of the whole reads.

Supplementary Figure 1


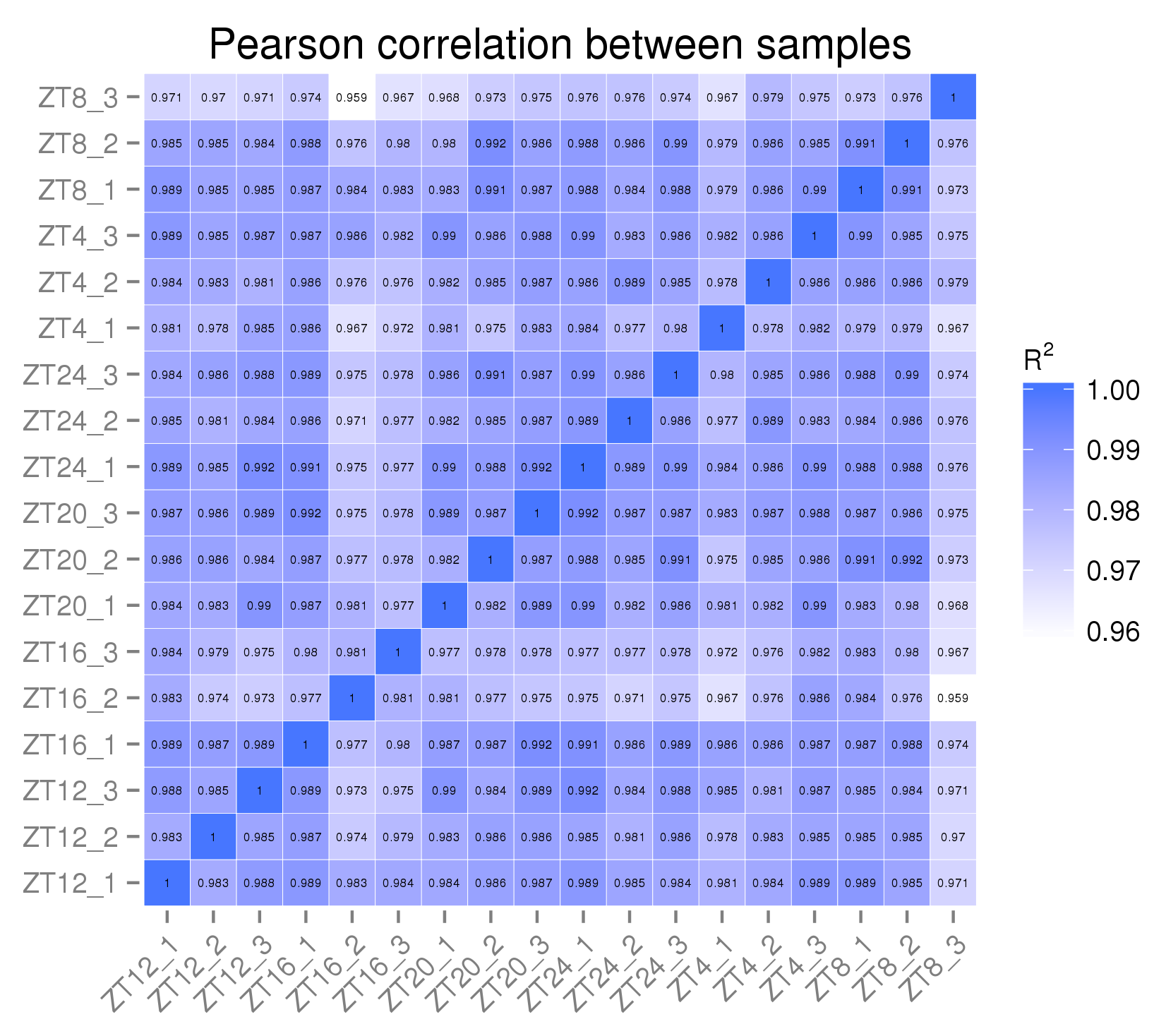


Fig. S1 Pearson correlation analysis of miRNA expression between samples. The abscissa and ordinate are log_10_ (TPM+1) of the sample. R^2^: The square of Pearson's correlation coefficient.
